# Whole-Genome Sequencing of Individuals from a Founder Population Identifies Candidate Genes for Asthma

**DOI:** 10.1371/journal.pone.0104396

**Published:** 2014-08-12

**Authors:** Catarina D. Campbell, Kiana Mohajeri, Maika Malig, Fereydoun Hormozdiari, Benjamin Nelson, Gaixin Du, Kristen M. Patterson, Celeste Eng, Dara G. Torgerson, Donglei Hu, Catherine Herman, Jessica X. Chong, Arthur Ko, Brian J. O'Roak, Niklas Krumm, Laura Vives, Choli Lee, Lindsey A. Roth, William Rodriguez-Cintron, Jose Rodriguez-Santana, Emerita Brigino-Buenaventura, Adam Davis, Kelley Meade, Michael A. LeNoir, Shannon Thyne, Daniel J. Jackson, James E. Gern, Robert F. Lemanske, Jay Shendure, Mark Abney, Esteban G. Burchard, Carole Ober, Evan E. Eichler

**Affiliations:** 1 Department of Genome Sciences, University of Washington, Seattle, Washington, United States of America; 2 Department of Human Genetics, The University of Chicago, Chicago, Illinois, United States of America; 3 Department of Medicine, University of California San Francisco, San Francisco, California, United States of America; 4 Veterans Caribbean Health Care System, San Juan, Puerto Rico, United States of America; 5 Centro de Neumología Pediátrica, San Juan, Puerto Rico, United States of America; 6 Department of Allergy & Immunology, Kaiser Permanente-Vallejo Medical Center, Vallejo, California, United States of America; 7 Children's Hospital and Research Center Oakland, Oakland, California, United States of America; 8 Bay Area Pediatrics, Oakland, California, United States of America; 9 San Francisco General Hospital, San Francisco, California, and the Department of Pediatrics, University of California San Francisco, San Francisco, California, United States of America; 10 Department of Pediatrics, University of Wisconsin, Madison, Wisconsin, United States of America; 11 Department of Medicine, University of Wisconsin, Madison, Wisconsin, United States of America; 12 Department of Bioengineering and Therapeutic Sciences, University of California San Francisco, San Francisco, California, United States of America; 13 Howard Hughes Medical Institute, Seattle, Washington, United States of America; Emory University School Of Medicine, United States of America

## Abstract

Asthma is a complex genetic disease caused by a combination of genetic and environmental risk factors. We sought to test classes of genetic variants largely missed by genome-wide association studies (GWAS), including copy number variants (CNVs) and low-frequency variants, by performing whole-genome sequencing (WGS) on 16 individuals from asthma-enriched and asthma-depleted families. The samples were obtained from an extended 13-generation Hutterite pedigree with reduced genetic heterogeneity due to a small founding gene pool and reduced environmental heterogeneity as a result of a communal lifestyle. We sequenced each individual to an average depth of 13-fold, generated a comprehensive catalog of genetic variants, and tested the most severe mutations for association with asthma. We identified and validated 1960 CNVs, 19 nonsense or splice-site single nucleotide variants (SNVs), and 18 insertions or deletions that were out of frame. As follow-up, we performed targeted sequencing of 16 genes in 837 cases and 540 controls of Puerto Rican ancestry and found that controls carry a significantly higher burden of mutations in *IL27RA* (2.0% of controls; 0.23% of cases; nominal p = 0.004; Bonferroni p = 0.21). We also genotyped 593 CNVs in 1199 Hutterite individuals. We identified a nominally significant association (p = 0.03; Odds ratio (OR) = 3.13) between a 6 kbp deletion in an intron of *NEDD4L* and increased risk of asthma. We genotyped this deletion in an additional 4787 non-Hutterite individuals (nominal p = 0.056; OR = 1.69). *NEDD4L* is expressed in bronchial epithelial cells, and conditional knockout of this gene in the lung in mice leads to severe inflammation and mucus accumulation. Our study represents one of the early instances of applying WGS to complex disease with a large environmental component and demonstrates how WGS can identify risk variants, including CNVs and low-frequency variants, largely untested in GWAS.

## Introduction

Complex genetic diseases are caused by many genetic and environmental factors. In the case of asthma, it has been estimated that genetic factors comprise 60% of the risk of developing this disease [1]–[4]. Genome-wide association studies (GWAS) have uncovered several strong links for asthma but, as with GWAS for other diseases, these variants explain only a small fraction of the genetic component [5]–[16]. Although GWAS have been powerful in identifying novel pathways associated with complex traits, including asthma, the small proportion of genetic risk explained by known associated single nucleotide polymorphisms (SNPs) suggests that other forms of genetic variation may play a substantial role in the development of disease. This conclusion is supported by a recent resequencing study that found evidence for a role of rare variants in the development of asthma [17]. Therefore, a more comprehensive survey of rare genetic variants in asthma is warranted.

Copy number variants (CNVs) are genetic variants that involve the deletion or duplication of greater than 50 bp of sequence [18]. CNVs can influence human phenotypes. Recently, rare and large CNVs have been reported to explain a substantial fraction (5%–15%) of the risk for certain severe complex disorders, including autism, mental retardation, and schizophrenia [19]–[23]. CNVs can be present at high frequency in the population and these variants are termed copy number polymorphisms (CNPs), and several CNPs have been strongly associated with a number of complex traits, especially immune-related diseases, including systemic lupus erythematous [24], [25], Crohn's disease [26], [27], HIV susceptibility [28], and psoriasis [29]. In addition, CNVs that involve deletions of glutathione S-transferase genes have been suggestively associated with asthma [30]–[34]. Also supporting the role of CNVs in the development of immune-related phenotypes is the finding that immunity genes are overrepresented in regions with common CNVs [35].

These reports point to the importance of assessing variation in CNPs for association to disease, yet this poses formidable technical challenges. Some CNPs are simple deletions or duplications and these variants are often in high linkage disequilibrium (LD) with SNPs [36]–[38]. However, many CNPs are found in complex regions of the genome with highly identical copies of paralogous sequence known as segmental duplications (SDs) [39], [40]. SD-associated CNPs often have many copy number states, are devoid of probes on SNP microarrays [37], and are not in LD with flanking SNPs [41], [42]. Therefore, due to these technical limitations, these variants have not been thoroughly tested for association to complex genetic traits. Recently, accurate estimation of copy number, even for highly complex regions, has been possible with whole-genome short-read sequencing [43], [44].

We hypothesized that CNVs, both common and rare, contribute to the etiology of asthma. We performed whole-genome sequencing (WGS) on 16 Hutterite individuals. Because of their reduced genetic and environmental heterogeneity, this population is ideal for the study of complex traits like asthma [45]. From these 16 genomes, we identified both single nucleotide variants (SNVs) and CNVs, which we tested for association in 1199 Hutterites. We identified several variants with potential association to asthma and attempted to replicate these results in 853 cases and 538 controls of Puerto Rican ancestry. Our study highlights the potential uses for WGS in the dissection of complex traits.

## Results

### Selection of individuals for WGS

The Hutterites are Anabaptists, who currently live on communal farms in the plains of the United States and Canada. They are descended from a small number of founders and their genealogy is known. From 1400 Hutterite individuals who are related in a 13-generation pedigree from 64 founders [46], [47], we selected 16 for WGS. We began by identifying three families within the extended Hutterite pedigree with an excess of individuals with asthma and three families from the extended pedigree with no individuals with asthma. From each of these six families, we selected one individual and their parents (when available) yielding three individuals with asthma and their parents (5/6 parents also had asthma) and three healthy controls and the parents of two of these individuals (3/4 parents did not have asthma). The trios were generally concordant in phenotype and allowed for additional support of identified variants through inheritance. We sequenced each of these 16 genomes to a coverage of 13-fold using an Illumina paired-end protocol (Table 1) as described previously [48]; all sequencing data are available in dbGaP (accession: phs000599.v1.p1).

**Table 1:** Summary of whole-genome sequencing.

	Phenotype	Sequence (Gb)	Coverage*	SNVs (millions)	Small CNVs#	Larger CNVs†
**1**	asthma	53	13.06	2.7	2683	445
father1	asthma	43	11.84	2.7	2737	466
mother1	asthma	47	12.47	2.8	2662	412
**2**	control	59	17.11	2.7	1886	459
father2	control	57	12.06	2.7	2556	454
mother2	symptoms	45	9.72	2.8	2534	425
**3**	control	63	16.28	2.8	1717	485
father3	control	51	10.51	2.7	2614	490
mother3	control	54	12.21	2.8	2716	407
**4**	asthma	69	15.25	2.8	3094	NA‡
father4	asthma	46	11.90	2.7	2672	351
mother4	asthma	36	10.72	2.7	2562	344
**5**	asthma	67	13.1	2.8	3267	383
father5	BHR§	39	13.28	2.8	2044	480
mother5	asthma	46	14.31	2.8	2727	415
**6**	control	58	14.40	2.8	3202	417
**ALL**	-	833	208.22	5.4	5852	1064
**MEAN**	-	52	13.01	2.8	2605	429

*Mean coverage of the genome (NCBI build 36) based on mapped reads.

# Identified from read-pair mappings.

† Identified with read-depth.

‡ Used as “reference” sample in the read-depth approach.

§ Bronchial hyperresponsiveness (BHR).

### Genetic variants identified from WGS

After obtaining raw sequencing reads, we applied pipelines optimized for CNV and SNV identification (Methods). We identified CNVs using both read-depth [44] and read-pair [49] signatures (Methods) as these approaches assess different parts of the CNV spectrum. In total, we identified 6916 CNVs in the 16 individuals (1064 were identified from read-depth and 5852 were identified from the read-pair approach) (Table S1). The CNVs identified from read-depth were larger (median size = 46 kbp) and more likely to be in SDs (885/1064 (83%) with >50% SD content) compared to those identified from read-pairs (median size = 292 bp; 1.0% in SDs) (Figure S1). We successfully targeted 2839 of the 6916 CNVs for validation with a custom microarray, confirming 1960 variants using comparative genomic hybridization (CGH), including 1137 CNVs not identified by the 1000 Genomes Project [50]. Of the 4077 variants that we could not target on the microarray, most (3209 of 4077) are deletions identified from paired-end mappings that are less than 1 kbp, the practical limit for CGH.

In addition, we identified 5.4 million SNVs and 576,000 indels in the 16 sequenced individuals (Methods; Table 1). To prioritize variants for validation and follow-up, we focused on those SNVs and indels predicted to be gene disruptive, not observed in the sequenced controls, and reported to be rare based on public databases available at the time (<5% allele frequency reported in the 1000 Genomes Project [51] or not present in dbSNP132). Applying these filters left us with 18 nonsense, 5 splice-site, and 23 frameshift mutations (Table S2). We validated 30 of these mutations (15 nonsense, 3 splice-site, and 12 frameshift) by PCR and Sanger sequencing.

**Figure 1:**
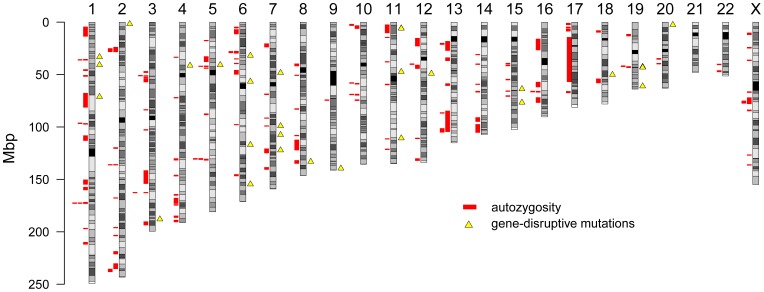
Overview of sequenced genomes. Ideograms of the autosomes and X chromosome are shown. Red bars represent segments of autozygosity observed in five individuals with asthma (parents of the sequenced trios) and yellow triangles represent asthma-specific gene-disruptive SNVs or indels. The regions of autozygosity in multiple cases on chromosomes 1q and 5q were also observed to be autozygous in at least one control individual.

Given the reduced genetic heterogeneity in the Hutterite population, we examined the data to see whether there were unexpected patterns of autozygosity (i.e., homozygosity by recent decent) in the individuals with asthma. We observed a number of autozygous segments longer than 1 Mbp, but there was no obvious overlap of segments in the individuals with asthma or overlap with predicted gene-disruptive mutations, arguing against a single recessive risk factor for the disease (Figure 1). Although this research was primarily motivated by the discovery of asthma genetic risk factors, the resource we have generated contributes to the catalog of rare and common genetic variation within the Hutterite population, including alleles that may be absent from other European populations [52]. Specifically, as previously reported [48], we identified 145,625 SNPs and 82,347 indels that were not in dbSNP build 135.

### Genotyping in additional individuals and comparison of CNV frequencies to another European-American population

**Figure 2:**
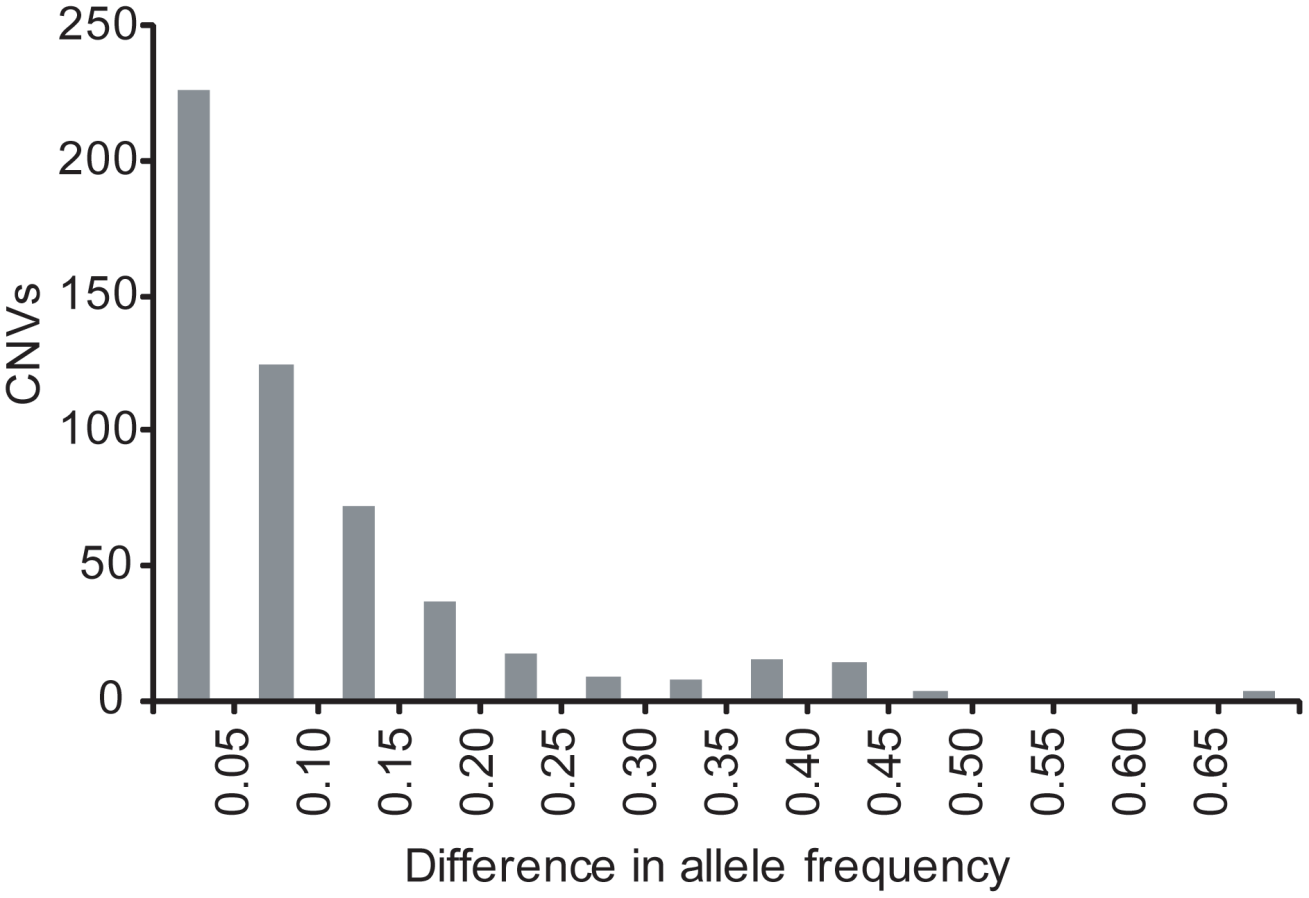
Comparison of CNV allele frequency between Hutterites and CEU. A histogram of the difference in allele frequency for the non-reference allele between Hutterites and the CEU individuals from the 1000 Genomes Project is shown. On the x-axis are bins for the absolute value of the non-reference allele frequency in the Hutterites minus the allele frequency in CEU. The y-axis represents the number of CNVs in each bin.

We used array CGH to genotype CNVs in additional Hutterite individuals. This genotyping was performed in two phases with 577 samples completed concurrently with WGS using a published microarray design [41] and 622 samples assessed with a microarray that targeted CNVs identified from WGS. Of the total 1199 individuals, 164 had diagnosed asthma based on the presence of symptoms, doctor's diagnosis, and bronchial hyperresponsiveness (BHR) [8], [53], 333 had BHR or asthma symptoms (but not both), 488 had neither asthma symptoms or BHR (controls), and 214 had unknown asthma status. It has been reported that SNPs have similar allele frequencies in Hutterites compared to other European-American populations (CEU) [54], and we sought to extend this result to CNVs. We compared the allele frequencies of the non-reference allele for 528 binary CNVs (i.e., simple deletions or duplications) and found the frequencies were correlated (r^2^ = 0.63) (Figure S2). In addition, there were very few variants with large differences in frequency between these two populations (Figure 2). Of the 18 CNVs with frequency differences greater than 0.4, five lie within introns of protein-coding genes.

### Association testing in Hutterites

**Figure 3:**
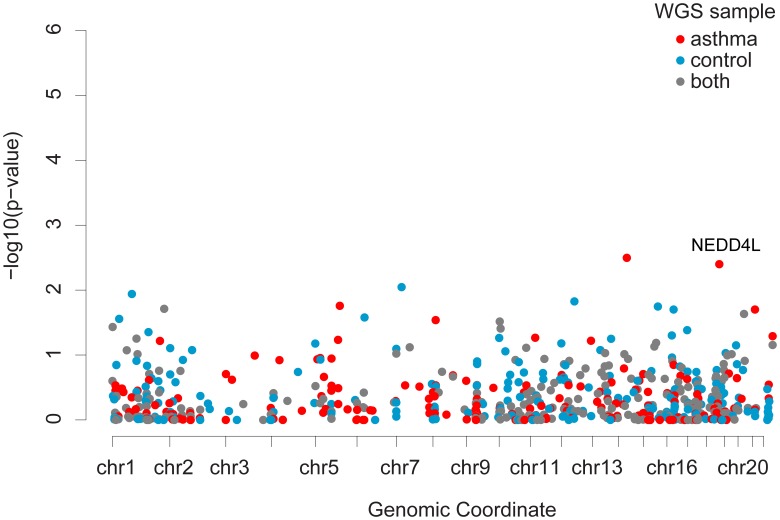
Association results of 593 CNVs to asthma. CNVs for association testing in the full Hutterite pedigree were identified in the 16 sequenced genomes. The points for these CNVs are colored based on the results of the whole-genome sequencing to represent whether the variant was observed in cases only (red), control individuals only (blue), or in both case and control individuals (gray). The genomic position is represented on the x-axis and the −log_10_(p-value) of the nominal association of each CNV to asthma in the full Hutterite pedigree is on the y-axis.

**Figure 4:**
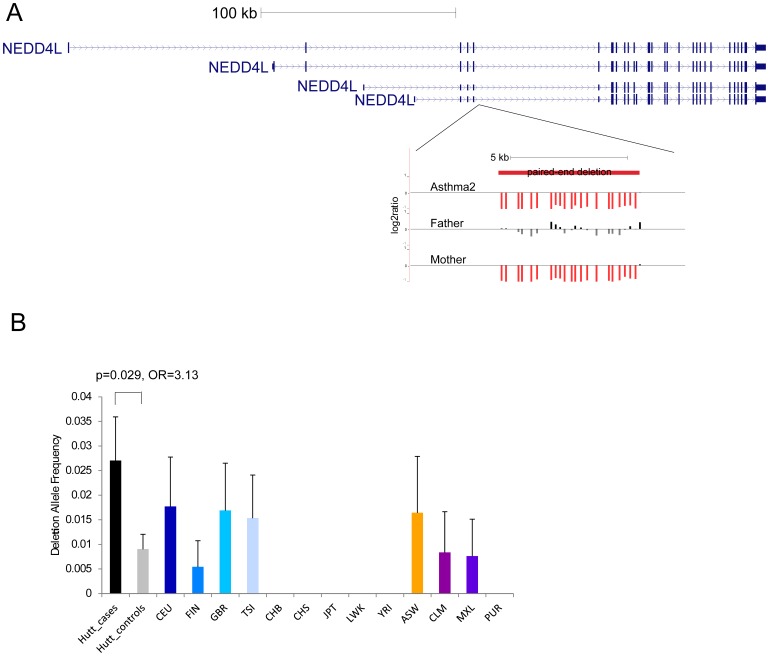
Identification of an intronic deletion in *NEDD4L* associated with asthma. (**A**) A representation of the location of the 6 kbp deletion. This deletion occurs in an intron shared by all reported transcripts of this gene (blue). The deletion was identified from paired-end sequence reads and validated by array CGH as shown for one of the sequenced trios. The log_2_ ratios of the probes in this region are shown as vertical bars with a log_2_ ratio of zero represented by the horizontal line. The red vertical bars in the child and mother indicate negative log_2_ ratios and confirm the deletion. (**B**) The frequency of this deletion in multiple populations is shown in the bar graph with the deletion allele frequency on the y-axis. The error bars represent the standard error on the allele frequency based on the binomial distribution. The Hutterite case (black) and control (gray) frequencies were determined by array CGH; the frequencies for the other populations are as reported by the 1000 Genomes Project.

We successfully tested 593 CNVs for association to asthma. We tested 204 CNVs with two alleles (i.e., simple deletions and duplications) for association using the MQLS test [55], [56]; for CNVs with more than two alleles, we used Wilcoxon rank-sum to compare the copy numbers between cases and controls (Figure 3). Although none of the associations for these CNVs would survive a multiple-testing correction, we identified 21 CNVs that were nominally associated with asthma in the full Hutterite sample (Table S3). A deletion in an intron of *NEDD4L* was present at 2.7% frequency in individuals with asthma and only at 0.9% in the controls (p = 0.03; Odds ratio (OR) = 3.13) (Figure 4).

We were able to successfully genotype 26 of the 30 validated gene-disruptive SNVs and indels in the full Hutterite sample and perform association testing [55], [56] (Table S4). The allele frequencies of these variants ranged from 0.15% to 11.0% in the Hutterites. Of these mutations, 13 were reported in the 1000 Genomes Project Phase 1 dataset [50] with allele frequencies between 0% and 16.7% in European populations (Table S2). There were 17 variants that were specific to the Hutterites and not observed in any individual from the 1000 Genomes Project Phase 1 [50]; these variants range from 0.15% to 10.1% allele frequency (median = 4.8%). These mutations included a stop-gain in an isoform of *DST* (encoding dystonin) and a predicted frameshift in a solute carrier expressed in *SLC24A1* (encoding a solute carrier); both of these genes are expressed in bronchial epithelial cells. From the genotyping of the 26 variants in the full Hutterite sample, we identified five variants with nominally significant associations to asthma in the Hutterites and had allele frequencies less than 0.05 in other populations of European ancestry, including the deletion in *SLC24A1*, and the allele frequencies of these variants range from 1%–6% in the Hutterites (Table 2).

**Table 2:** SNVs and indels nominally associated with asthma in the Hutterites.

chr	Pos*	dbSNP	gene	mutation	CEU freq#	Hutterite freq†	p†	OR‡
3	187944218	rs76438938	KNG1	R376X	0.024	0.05	0.019	2.34
6	154609555	rs34427887	OPRM1	R401X	0.000	0.05	0.022	1.66
15	63733326	.	SLC24A1	L1053fs	0.000	0.04	0.013	2.38
18	50134887	rs17292725	STARD6	R19X	0.024	0.04	0.041	1.78
19	43072247	.	WDR87	E1263X	0.000	0.01	0.036	2.03

*Position in NCBI build 36.

# Based on the 1000 Genomes Project Phase 1 [50].

† Corrected for relatedness as previously described [55].

‡ Odds ratio (OR).

### Extension to additional populations

None of the variants tested in the Hutterites met genome-wide significance due to limited power; therefore, we sought to extend our results to additional populations. Using a breakpoint PCR assay (Methods), we were able to genotype the *NEDD4L* deletion in an additional 736 cases and 755 controls of European ancestry, 755 cases and 742 controls of Puerto Rican ancestry, and 1052 cases and 747 controls of African American ancestry. We did not observe a significant association with asthma when combining the results of the replication studies (one-tailed p = 0.073; OR = 1.55 (95% CI: 0.90–2.71)) (Table 3).

**Table 3:** Association of intronic deletion in *NEDD4L* with asthma in additional populations.

Study	Ancestry	N Cases; N Controls	Case Freq	Control Freq	p	OR*
Chicago	European American	177; 219	0.014	0.000	0.051	Inf
Freiburg	German	370; 364	0.015	0.015	0.97	0.98
COAST	European American	189; 172	0.019	0.015	0.64	1.27
GALAII	Puerto Rican	755; 742	0.001	0.000	0.50	Inf
SAGE	African American	1052; 747	0.004	0.003	0.22	1.6
**COMBINED** #		**2543; 2244**			**0.073**	**1.69**

*Odds ratio (OR).

# Combined association statistics were obtained using the Cochran–Mantel–Haenszel test.

Because we were motivated to identify genetic risk variants for asthma that would have been missed by GWAS, it is not surprising that many of the variants we identified are at low or moderate allele frequency. Therefore, to extend these results to an additional population, we performed targeted resequencing of seven candidate genes with rare gene-disruptive variants identified in our WGS data that also had other evidence for a role in asthma (e.g. near GWAS association peak). In addition, we had capacity in our resequencing design to include nine genes from other sources, including those genes affected by CNVs unique to individuals with asthma in the NHLBI Exome Sequencing Project that had additional biological or genetic support of a role in asthma (Krumm et al. unpublished data). We used molecular inversion probes (MIPs) to perform targeted resequencing [57]–[59] of 16 loci (95 kbp) (Table S5) in 853 cases and 538 controls of Puerto Rican ancestry [60], [61]. We chose to focus our replication efforts individuals of Puerto Rican descent have the highest incidence of asthma and most severe disease of any ethnic group in the United States [62], [63].We identified 522 SNVs and 13 indels that altered protein sequence, and of these, 11 SNVs lead to the gain of a stop codon and 12 indels result in a predicted frameshift in the protein open reading frame (Table S6). For each targeted gene, we used SKAT-O [64], corrected for local ancestry, to determine whether there was a potential involvement of coding variation with asthma (Table S7). Specifically, we included all nonsense, all coding indels, and any missense, putative splice-site alteration with a GERP score [65] greater than three. In addition, we filtered out variants with an allele frequency >0.05 in the groups consisting of individuals from the Americas, Africa, or Europe in the 1000 Genomes Project [50]. The mean GERP score for the observed nonsense mutations was 2.5, so we selected 3 as a threshold for missense and splice-site mutations.

In *SLC24A1*, where we had observed an indel frameshift nominally associated with asthma in the Hutterites (p = 0.01, OR = 2.38), we observed 23 rare missense mutations and one nonsense mutation in the Puerto Rican individuals (Table S7). This nonsense mutation was observed in a single individual with asthma and occurs in the first coding exon of the gene. This gene was not significant by SKAT-O for the presence of genetic variation influencing the development of asthma in this population. Interestingly, in *IL27RA* we observed a nominally significant SKAT-O result (p = 0.004) when we tested the effects of the five missense mutations with GERP greater than three and one insertion that results in a frameshift. This gene was targeted because of the presence of CNVs discovered from exome sequencing and specific to individuals with lung disease. Upon further examination of the variants in this gene, we found that controls were more likely to carry a conserved missense or indel in *IL27RA* than cases (2.0% of controls vs. 0.23% of cases) (Figure S3), and the association with SKAT-O remained whether or not we included ancestry in the model.

## Discussion

We explored a new approach for dissecting the genetic risk factors of asthma. WGS is currently neither affordable nor feasible on the large number of individuals required to have sufficient power for detecting associations with asthma. Therefore, we developed an approach involving the sequencing of a small number of index individuals and then using these data to test for association in additional individuals. This tactic was especially suited to a population such as the Hutterites. Because the current population of Hutterites is descended from a small number of founding individuals, genetic heterogeneity is reduced. Therefore, the variants discovered by sequencing a subset of individuals will represent a large fraction of the total genetic variation in the population than if the sequenced individuals were drawn from a more genetically diverse population. Accordingly, we identified and genotyped 14 gene-disruptive variants that were not observed in the 1000 Genomes Project yet have allele frequencies between 0.15% and 10.1% in the Hutterites.

We performed follow-up resequencing of genes identified in WGS of Hutterite individuals as well as in exome sequencing in Puerto Rican individuals to determine if these results could be replicated and extended to another population. We did not observe a significant enrichment of disruptive or rare protein-coding mutations across the selected genes in the cases compared to controls. In addition, we observed no association in the genes selected from the sequencing analysis in the Hutterites, and this result may be due spurious associations in the Hutterites or to heterogeneity in rare variant between populations. For *IL27RA*, we observed a nominally significant increase of rare mutations in control individuals. IL27 is thought to be an inhibitory cytokine, which should function through its receptor to reduce inflammation [66]. If these mutations are indeed functional, then perhaps they increase or change the activity of *IL27RA*. Interestingly, the five conserved missense mutations occurred in predicted extracellular fibronectin 3 domains (Figure S3), which may function in protein-protein interactions. In addition, IL-27 is a member of the IL-6/IL-12 family of cytokines [66], and rare variants in *IL12RB1* have been previously implicated in asthma [17], suggesting that this family of cytokines and receptors should be the target of additional studies.

We observe some interesting associations between specific variants and asthma, but none of these associations would be significant with multiple-testing correction suggesting the need for larger sample sizes. These variants include a deletion in the intron of *NEDD4L*, which is a gene implicated in asthma by other genetic and functional evidence. However, this deletion is quite rare in the populations tested with allele frequencies ranging from 0.07% in Puerto Ricans to 0.6% in Hutterites (Table 3), which severely limits our power to find a significant association. *NEDD4L* is expressed in bronchial epithelial cells [67] and a conditional knockout of this gene in mice leads to a cystic fibrosis-like phenotype including inflammation and mucus overproduction [68], also features of asthma, suggesting that this gene plays an important role in the lung. In addition, *NEDD4L* is under a linkage peak on chromosome 18 for asthma symptoms identified in the Hutterites [53]. We also observed a nominally significant association with a frameshift in *SLC24A1*, a gene also expressed in bronchial epithelial cells [67] and located near an association signal from GWAS on chromosome 15 [6]. In addition, we observed a nonsense mutation in a single Puerto Rican case. Finally, we observed an increase in rare mutations in controls in *IL27RA*. Taken together, these results highlight the need for large sample sizes to find “genome-wide” significant associations of rare variants.

We used WGS to develop a catalog of variants that could then be further tested for association. Obviously, this approach is by no means comprehensive of all variants that influence the development of disease present in this population or even in the individuals sequenced. Increased sensitivity would require higher depth-of-coverage, longer sequence read lengths, and larger insert libraries. Interestingly, similar to a recent report on bipolar disorder in the Amish [69], the difficulty in identifying variants strongly associated with asthma in the Hutterites may point to the genetic heterogeneity of this disease. In addition, our study points to the difficulty of genotyping complex CNPs in a large number of individuals. We used custom microarrays in order to assess as many variants as possible; however, there are many CNPs that do not perform well on microarrays due to small size or the limited dynamic range of array CGH. This emphasizes the need for new technologies that can be used to genotype CNPs in a high-throughput and low-cost manner. Our study represents one of the early instances of applying WGS to understanding a common, complex disease. Given that deep WGS is not yet affordable for the thousands of individuals required, the approach we outline here may be applicable to other diseases.

## Methods

All individuals consented, and the project was approved by the institutional review boards of the University of Chicago, the University of Washington, and the University of California, San Francisco.

### DNA samples

The 16 individuals for WGS were selected from a 13-generation Hutterite pedigree: eight individuals diagnosed with asthma, six healthy controls, one with BHR, and one with asthma symptoms. To select individuals for sequencing, we examined families within the Hutterite pedigree that were descended from no more than six individuals. We then identified those families that had enrichment or depletion of asthma compared to other such families. Finally, we selected trios, where possible, from six families for sequencing: three enriched for asthma and three with no individuals with asthma. There is some overlap in the membership of these six families. Fifteen of these individuals are the same as previously published [48]. The 1199 individuals assessed with array CGH and the 689 individuals (171 cases and 518 controls) genotyped for selected SNVs and indels are drawn from the same sample of Hutterites. We resequenced DNA isolated from the whole blood of 1391 individuals of Puerto Rican ancestry (853 cases and 538 controls) from the Genetics of Asthma in Latino Americans (GALA II) studies [61].

### Whole-genome sequencing (WGS)

WGS has been previously reported [48]. Briefly, we generated libraries using 1–3 ug of genomic DNA. We sequenced these samples to an average effective coverage of 13X using Illumina paired-end reads (PE51 and PE101) on an Illumina Hi-Seq 2000. Sequencing data are available under dbGaP accession number phs000599.v1.p1 (http://www.ncbi.nlm.nih.gov/projects/gap/cgi-bin/study.cgi?study_id=phs000599.v1.p1).

### SNV identification and validation

We identified SNVs from the WGS data as previously described [48]. Briefly, we aligned sequence reads to the human reference genome (NCBI build 36) using the software BWA [70]. We processed the resulting BAM files with Picard tools to remove reads due to PCR duplicates, GATK to realign reads around candidate indels and recalibrate base quality scores [71]. We used multisample calling in GATK to identify SNVs and indels, and we filtered these variants using variant quality score recalibration (VQSR) in GATK [72]. We used a VQSR threshold of 2.30 (99% of known high-quality SNPs identified) to generate a final list of SNVs. Indels were filtered using GATK recommendations [71]. As previously reported, we determine the false negative rate for heterozygous SNPs by comparing the genotypes obtained from WGS to those from Affymetrix 6.0 SNP microarrays that were available for 15 of the 16 individuals. The false negative rate ranged from 1.53%–2.90% [48]. We annotated all variants using Annovar [73] to determine which mutations were located in coding sequence, and we then annotated all coding variants more thoroughly using SeattleSeq (http://snp.gs.washington.edu/SeattleSeqAnnotation129/). We selected SNVs and indels for Sanger sequence validation that were observed in individuals with asthma only and were nonsense, splice-site, or frameshifting indels. In addition, we required that variants have an allele frequency below 0.05 in the 1000 Genomes Pilot dataset [51]. We designed primers flanking these variants using batch Primer3 [74]. Each targeted region was amplified with PCR and sequenced in both directions with Sanger sequencing by GENEWIZ (www.genewiz.com). PCR and sequencing was performed on the individual(s) harboring the variants of interest as well as their parents (in the case of children in trios) or their child (in the case of parents) and any individual lacking a genotype call from WGS. The resulting sequences were aligned to the reference genome using Sequencher (http://genecodes.com/) and manually inspected for the presence of the expected SNV or indel.

### CNV discovery

We used two complementary methods to identify CNVs from WGS data. First, we identified CNVs by an increase or decrease in sequencing read-depth as previously described [43], [44]. Briefly, we aligned the sequencing reads to all possible mappings in the human reference genome using the software mrsFAST [75]. We analyzed the relative read-depths of fifteen of the sequenced samples against the sixteenth sample to identify CNVs as well as determining the copy numbers of previously reported CNVs based on absolute read-depth using previously described methods [44]. We compared these copy numbers to those of the 45 CEU individuals in the pilot phase of the 1000 Genomes Project [51] to identify variants where we observed an outlying individual among the sequenced Hutterites. We filtered out CNVs where all the copy numbers of the Hutterite individuals were between 1.5 and 2.5 as non-variant. We cataloged 389 CNVs where there was an apparent difference between either the cases or controls and the 45 CEU genomes (Wilcoxon rank-sum p< = 0.01), CNVs where there was a single Hutterite outlier with an absolute value of z-score great than two compared the copy number distribution of the 45 CEU genomes, and CNVs where both the cases and controls appeared different from the 45 CEU genomes (Wilcoxon rank-sum p< = 0.01 in one group and Wilcoxon rank-sum p<0.1 in the other). These CNVs were included in the validation experiments outlined below. We also used read-pair methods to identify CNVs [49]. Briefly, we aligned the paired-end reads to all potential mappings in the reference genome using the aligner mrFAST [43]. We then identified putative deletions simultaneously in the 16 genomes using the programs VariationHunter [49] and CommonLaw [76], which identify clusters of paired-end mappings where the reads mapped further apart than expected based on the insert sizes of the libraries.

### CNV validation

We designed a custom microarray using the Agilent 2X400K SurePrint G3 Human CGH Microarray Platform with 400,000 probes targeted to the CNVs identified with both the read-depth and read-pair approaches. In addition, 3000 standard Agilent normalization probes located throughout the genome and five replicates of 1000 probes were included. We performed the sample labeling and hybridization using Agilent recommended protocols. We hybridized fluorescently labeled DNA from each of the 16 individuals along with a reference individual (CEU female, NA12878) to this microarray. Microarray data were extracted from the image files with Agilent FE software using a modification of the CGH-105_Dec08 protocol. The microarray data were normalized to the 3000 Agilent normalization probes located throughout the autosomes. For each targeted CNV, we computed the median log2 ratio of all probes in the putative variant. We considered CNVs to be validated if the absolute value of the log2 ratio was greater than 0.5.

### CNV genotyping

We performed CNV genotyping in two phases. The first phase used a published array design targeted to known CNPs in the genome [41], and the second phase of genotyping used a modified version of this design that targeted validated CNVs identified in WGS. Both microarrays were Agilent 4X180K SurePrint G3 Human CGH Microarrays with 3000 standard Agilent normalization probes located throughout the genome and five replicates of 1000 probes. Samples were fluorescently labeled and hybridized as described above using NA12878 as a reference sample. Microarray data were processed with Agilent FE software and normalized to the 3000 Agilent control probes. We implemented several quality control metrics for the resulting array CGH data. We required that all metrics calculated by Agilent FE met the Agilent suggested thresholds. Additionally, to exclude noisy hybridizations, we required that the derivative log ratio (DLR) score was below 0.24. We repeated samples that failed these criteria up to two additional times. Finally, to guard against sample mix-up we removed any samples where the microarray data was not concordant with the reported gender.

We used these microarray data to genotype CNVs as previously described [41], [77]. Data from different microarray designs were considered separately for copy number genotyping to avoid batch effects. Briefly, we computed the median log2 ratio, median test sample signal, and median reference sample signal for all probes within a CNV. To determine the performance of each CNV on the microarray, we calculated the ratio of the coefficient of variation of the median signal intensity of the test sample signal and the coefficient of variation of the median signal intensity of the reference sample signal. This value, which we term rCV, will be large when there is variation among the test samples and reproducibility in the reference sample suggestive of a polymorphic variant. A low rCV suggests that the CNV is either not variant in the samples tested or performs poorly on the microarray leading to a lack of reproducibility in the reference signals across hybridizations. We considered CNVs with rCV>1.4 (as previously published [41]) to be variant and well performing on the microarray, and we restricted our analysis to these variants.

For the well-performing CNVs in phase 1 and phase 2, we estimated copy number using a combination of log2 ratio and signal intensity data. The median log2 ratios and signal intensity values were clustered across samples into discrete copy number classes when possible as previously described [41], [77], [78]. For each CNV, we used two slightly different methodologies to fit the data integer copy numbers: one methodology simultaneously fits the log2 ratios and signal intensities and the other fits log2 ratios only. To evaluate which method performed more accurately, we calculated the correlation of the copy numbers estimated from array CGH to those estimated from sequencing read-depth for individuals from the 1000 Genomes Project (N = 47 for set 1 and N = 40 for set 2) [44], [50], who we also assessed on our custom microarrays. We selected the copy numbers for each CNV from the method that was most correlated with the copy numbers from WGS, or we selected the method that yielded integer copy number states. Finally, we only considered CNVs for further analysis if the resulting copy numbers were correlated with those from sequencing read-depth with a correlation coefficient (r) of at least 0.65.

For CNVs that could not be fitted to integer copy number states, we estimated the copy number based signal intensity data. We first determined the signal intensity corresponding to a single copy and then used this value to estimate the copy number of the reference sample for each CNV. Then, we estimated the copy number of each test sample from the copy number of the reference sample and the log2 ratio. To test whether the estimated copy numbers were accurate, we compared to the 1000 Genomes Project individuals as described above and excluded CNVs with r<0.65 to sequencing read-depth copy numbers.

### Association testing

As an initial test for association of the genotyped CNVs with asthma, we performed Wilcoxon rank-sum tests between the copy numbers of cases and controls. For simple deletions and duplications where we could assign allelic copy numbers, we performed association testing correcting for the relatedness of the individuals [55]. We genotyped rare, asthma-specific, gene-disruptive SNVs and indels in the full sample of Hutterites using the Sequenom iPLEX system. We were able to design assays and genotype 26 of the 30 variants. Of the remaining four variants, we successfully genotyped one using TaqMan genotyping assays. We performed association testing of the genotyped variants correcting for relatedness of the individuals as previously described [55]. All reported p-values are nominal and not corrected for multiple testing.

### Molecular inversion probe (MIP) resequencing

To test genes identified by WGS in another population, we designed MIPs to capture the coding sequence of 15 genes for resequencing as previously described [57]. We designed 1240 70-mer MIPs that overlap and alternate strands to capture a total of 85 kbp of sequence. We pooled equimolar amounts of each MIP with 50-fold excess added for MIPs with low design scores or high (>65%) or low GC content of the captured sequence. The concentration of 1X probes was 0.0096 µM in the final pool. We tested our pooled MIPs on DNA from 24 individuals from the HapMap Project [79]. From these results, we determined that an additional 171 MIPs showed inefficient capture, so we added 50-fold excess of these probes as well. The final pool of MIPs was phosphorylated with 100 units of T4 PNK (New England Biolabs).

Capture experiments were carried out as follows [79]. We performed capture using 100 ng of genomic DNA for each individual that was mixed with 4.3×10^−5^ pmole of each 1X MIP of the final pool, 0.4 units of StoffelTaq (Applied Biosystems), 1 unit of Ampligase (Illumina), and 8 pmole dNTPs and then denatured at 95°C for 10 minutes and incubated at 60°C for 23 hours. The resulting product was treated with 10 units EXOI (New England Biolabs) and 50 units of EXOIII (New England Biolabs) to remove unreacted (non-circularized products). The captured product was amplified with PCR using barcoded primers under the following conditions to produce the final libraries. Five microliters of exonuclease MIP captured product was mixed with 25 µM of 2X iProof master mix, 0.025 nmole each of forward and reverse primers in a 50 µL reaction, and amplified. 96 barcoded samples were pooled and cleaned with AMPure magnetic beads (Beckman Coulter), and then, four cleaned pools were pooled for sequencing on an Illumina HiSeq with 100 bp paired-end reads (384 individuals per lane).

### Targeted resequencing analysis

We processed the sequencing reads from the MIP capture experiments as follows. First, we split the fastq reads by barcode to generate files for each individual. We aligned the sequencing reads for each individual to the human reference genome (build hg19) using BWA [70]. We filtered for reads that were properly paired, mapped to expected locations, and had the expected insert size. We removed sequence corresponding to the MIP targeting arms. We realigned reads around putative indels with GATK [71] and clipped reads to 56 bases to avoid overlap between forward and reverse reads from the same capture event. We excluded individuals who had less than 25,000 total reads from variant calling.

We identified SNVs and indels with GATK [71]. We required that SNV genotypes have a genotype quality greater than 20 and a depth-of-coverage of at least eight; in addition, we required that the average allele balance for heterozygous genotypes of an SNV was less than 0.75. For indel genotypes, we required a genotype quality of at least 50 in addition to a depth-of-coverage of at least eight and an allele balance less than 0.75. After applying these genotype filters. We annotated the filtered variants with SeattleSeq (http://snp.gs.washington.edu/SeattleSeqAnnotation129/). We performed association analysis with SKAT-O [64] at the level of reported transcript first by including all protein-altering mutations (missense, nonsense, splice-site, and indels) and second by including all nonsense and rare coding indels plus rare missense and splice-site mutations with a GERP [65] score greater than three. Rare was defined as less than 0.05 allele frequency in 1000 Genomes individuals from the Americas, Africa, and Europe [50]. Briefly, SKAT-O performed an aggregate analysis of variant sets (variants within a specific transcript, in this case) to determine whether the patterns of variation are different between cases and controls [64]. We weighted all variants equally and all association analyses were corrected for genomic and locus-specific African, European, and Native American ancestry by including these estimates as covariates. Variants with greater than 15% missing data were excluded. Nominal p-values are reported for each transcript in addition to conservative Bonferroni corrected p-values for 29 transcripts and two tests (Table S7). Genomic ancestry was estimated using ADMIXTURE [80] and local ancestry was estimated using LAMP-LD [81], [82], both from SNP-microarray data from the Axiom LAT1 array (World Array 4; Affymetrix, Santa Clara, CA) as previously described [83]. Local ancestry for a gene was computed as the average ancestry across genotyped SNP between the transcription start and stop site.

## Supporting Information

Figure S1 **Size distributions of CNVs identified from WGS.** A histogram of CNVs by size for two approaches to CNV detection is shown with the size bins on the x-axis and the count of CNVs on the y-axis. A larger number of CNVs were identified from the paired-end mapping data (red) and these tended to be smaller. CNVs identified using read-depth information (blue) were larger and more likely to be in segmental duplications.
(TIF)

Figure S2 **Correlation of allele frequency between Hutterites and CEU for genotyped CNVs.** The scatter plot shows the allele frequency for the non-reference allele of binary CNVs genotyped in the Hutterites (x-axis) and CEU individuals from the 1000 Genomes Project (y-axis). The y = x line is also plotted.
(TIF)

Figure S3 **Distribution of mutations in** ***IL27RA*** **.** A diagram of *IL27RA* with its reported domains is shown. On the top are the conserved missense (yellow) and frameshift (red) mutations observed in the controls with the number of controls carrying that mutation. On the bottom are the mutations in cases and the number of cases carrying each mutation.
(TIF)

Table S1 **CNVs identified by WGS.** Coordinates are in build hg18 of the human reference genome. CNVs are annotated by the method used to identify them (RD = read-depth; RP = read-pair). CNVs are annotated by whether they were observed in the 1000 Genomes Pilot dataset and whether they were validated by array CGH.
(XLSX)

Table S2 **Gene-disruptive SNVs and indels observed in the genomes from individuals with asthma.** Coordinates are in build hg18 of the human reference genome. Hutterite frequency and p-value for association to asthma were determined by genotyping variants in a larger sample of Hutterites (N∼1400). Frequencies for the 1000 Genomes Project European populations are from the Phase I dataset (CEU = individuals of Northern and Western European ancestry living in Utah; FIN = individuals from Finland of Finnish ancestry; GBR = individuals of British ancestry living in England and Scotland; TSI = individuals of Toscani ancestry living in Italy).
(XLSX)

Table S3 **CNVs nominally associated with asthma in Hutterites.** Coordinates are in build hg19 of the human reference genome. Variants are annotated based on whether they were observed in individuals with asthma, controls, or both in the WGS and based on the type of CNV (del = deletion, dup = duplication, complex = more than two alleles). “Set” refers to the batch of microarrays where the CNV was genotyped and “test” refers to the association test performed.
(XLSX)

Table S4 **Summary of genotyping results for gene-disruptive SNVs and indels.** Coordinates are in build hg18 of the human reference genome. Association analysis was performed correcting for the relatedness of the individuals. The number of individuals in each phenotypic class successfully genotyped for each assay is represented in the “N Cases/N Controls/N Unknown” column.
(XLSX)

Table S5 **Gene selected for targeted resequencing.**
(XLSX)

Table S6 **SNVs and indels identified in targeted resequencing.** Coordinates are in build hg19 of the human reference genome. MAF = minor allele frequency; HWE = Hardy Weinberg Equilibrium p-value; OR = odds ratio; VALIDATION_STATUS = results of Sanger sequencing validation.
(XLSX)

Table S7 **Results of transcript level association.** Results are shown for SKAT-O test using either all protein-altering variation or filtering for rare variants with a GERP score >3.
(XLSX)
