# Supplementary figures and images for: Whole-Genome Sequencing of Individuals from a Founder Population Identifies Candidate Genes for Asthma
Source: PLoS One. 2014 Aug 12;9(8):e104396. doi: 10.1371/journal.pone.0104396 (PMC4130548; doi:10.1371/journal.pone.0104396)

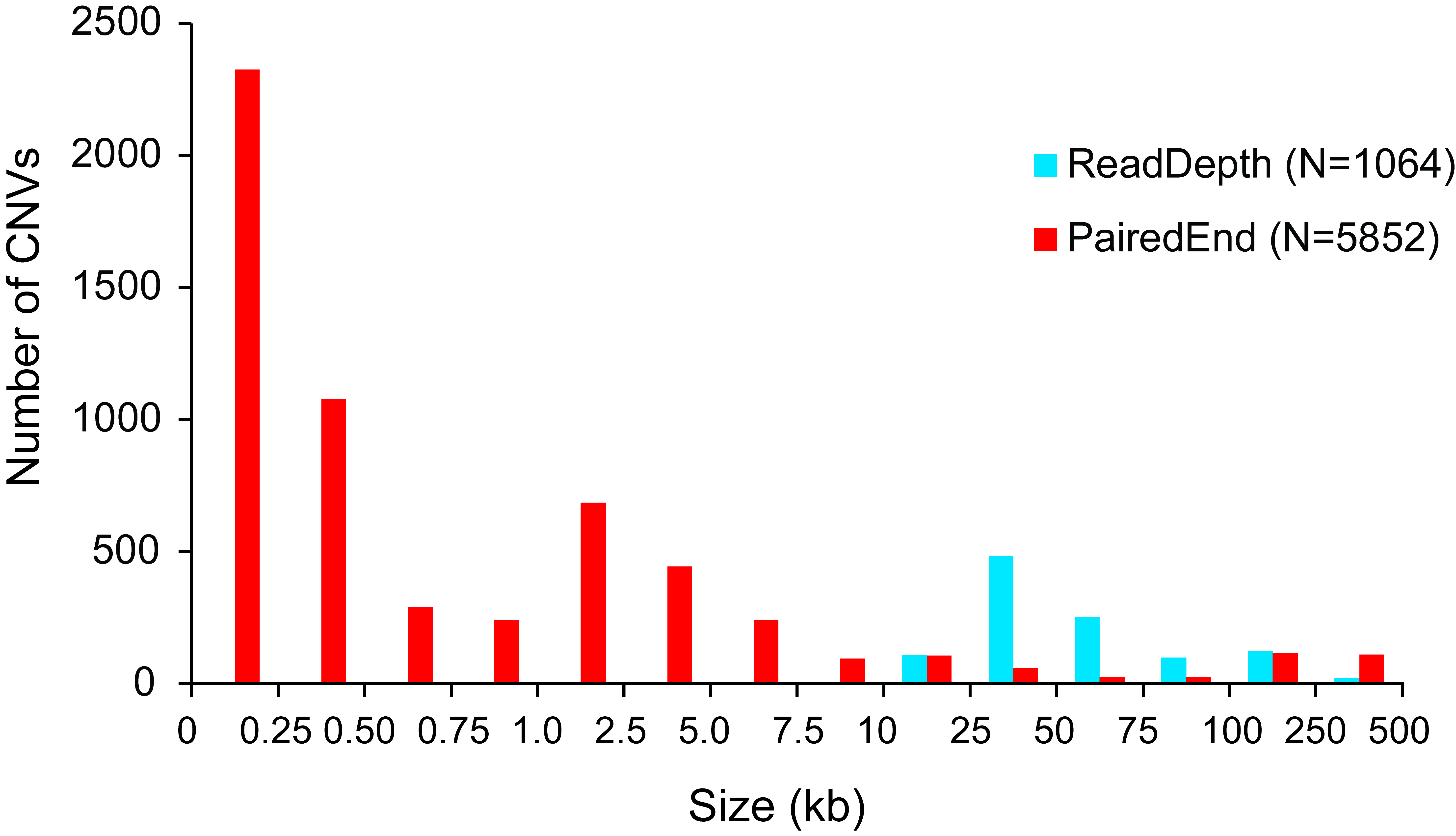

Supplement: Figure S1 — Size distributions of CNVs identified from WGS. A histogram of CNVs by size for two approaches to CNV detection is shown with the size bins on the x-axis and the count of CNVs on the y-axis. A larger number of CNVs were identified from the paired-end mapping data (red) and these tended to be smaller. CNVs identified using read-depth information (blue) were larger and more likely to be in segmental duplications. (TIF) [file pone.0104396.s001.tif]

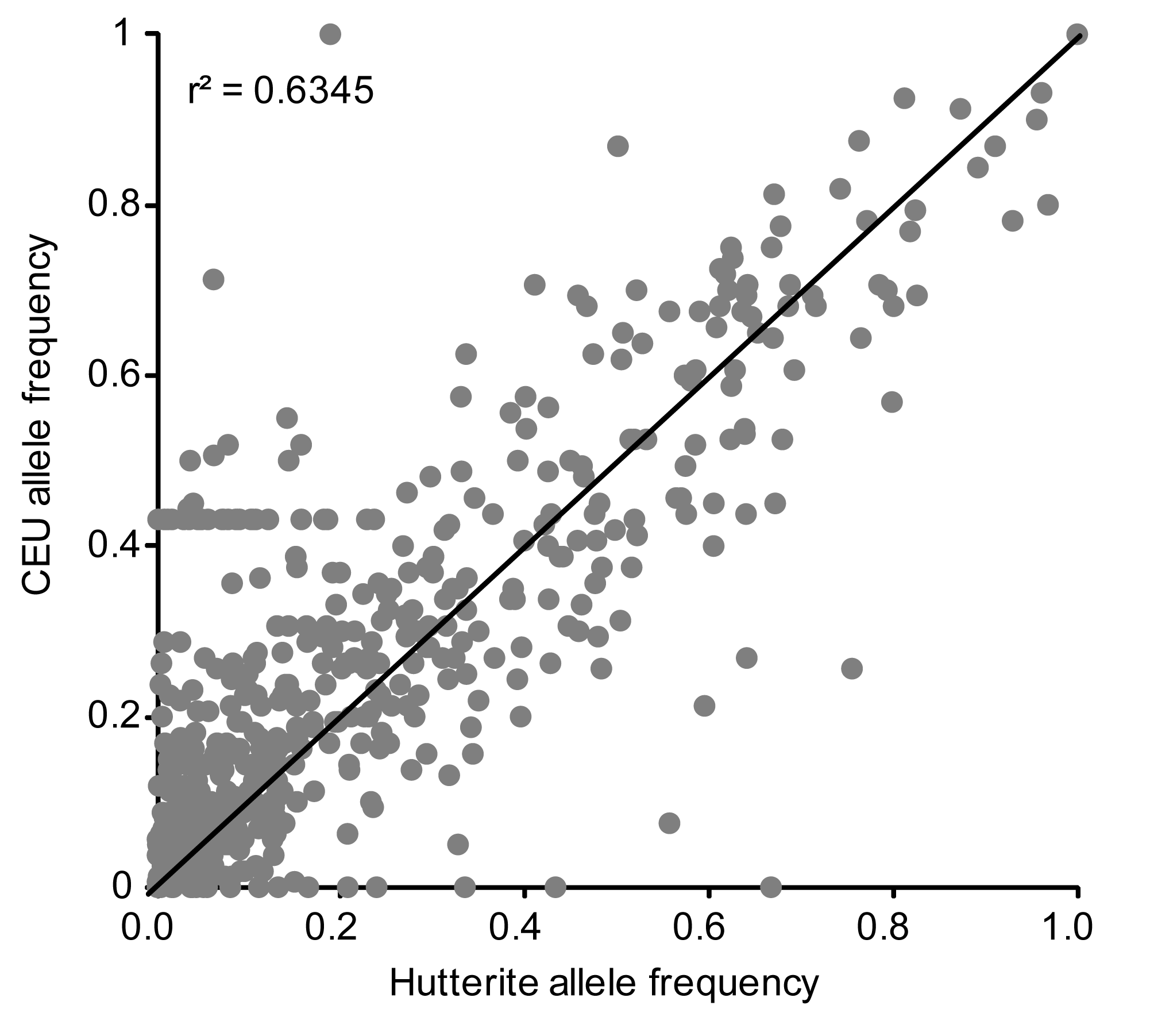

Supplement: Figure S2 — Correlation of allele frequency between Hutterites and CEU for genotyped CNVs. The scatter plot shows the allele frequency for the non-reference allele of binary CNVs genotyped in the Hutterites (x-axis) and CEU individuals from the 1000 Genomes Project (y-axis). The y = x line is also plotted. (TIF) [file pone.0104396.s002.tif]

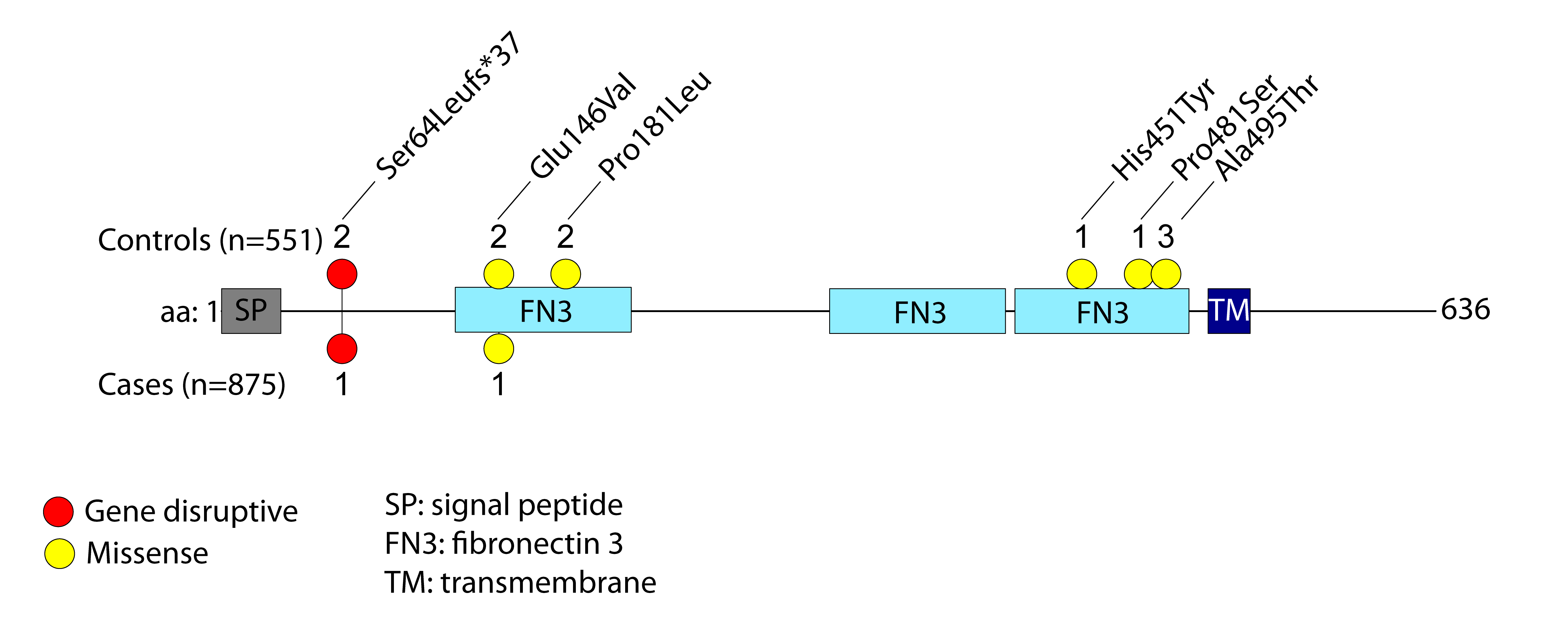

Supplement: Figure S3 — Distribution of mutations in IL27RA . A diagram of IL27RA with its reported domains is shown. On the top are the conserved missense (yellow) and frameshift (red) mutations observed in the controls with the number of controls carrying that mutation. On the bottom are the mutations in cases and the number of cases carrying each mutation. (TIF) [file pone.0104396.s003.tif]
